# Impact of input data (in)accuracy on overestimation of visible area in digital viewshed models

**DOI:** 10.7717/peerj.4835

**Published:** 2018-05-21

**Authors:** Ondřej Lagner, Tomáš Klouček, Petra Šímová

**Affiliations:** Department of Applied Geoinformatics and Spatial Planning, Faculty of Environmental Sciences, Czech University of Life Sciences Prague, Praha - Suchdol, Czech Republic

**Keywords:** LiDAR, Spatial uncertainty, Digital surface model, Viewshed, Data quality

## Abstract

Viewshed analysis is a GIS tool in standard use for more than two decades to perform numerous scientific and practical tasks. The reliability of the resulting viewshed model depends on the computational algorithm and the quality of the input digital surface model (DSM). Although many studies have dealt with improving viewshed algorithms, only a few studies have focused on the effect of the spatial accuracy of input data. Here, we compare simple binary viewshed models based on DSMs having varying levels of detail with viewshed models created using LiDAR DSM. The compared DSMs were calculated as the sums of digital terrain models (DTMs) and layers of forests and buildings with expertly assigned heights. Both elevation data and the visibility obstacle layers were prepared using digital vector maps differing in scale (1:5,000, 1:25,000, and 1:500,000) as well as using a combination of a LiDAR DTM with objects vectorized on an orthophotomap. All analyses were performed for 104 sample locations of 5 km^2^, covering areas from lowlands to mountains and including farmlands as well as afforested landscapes. We worked with two observer point heights, the first (1.8 m) simulating observation by a person standing on the ground and the second (80 m) as observation from high structures such as wind turbines, and with five estimates of forest heights (15, 20, 25, 30, and 35 m). At all height estimations, all of the vector-based DSMs used resulted in overestimations of visible areas considerably greater than those from the LiDAR DSM. In comparison to the effect from input data scale, the effect from object height estimation was shown to be secondary.

## Introduction

Defining the visibility of objects in the landscape has been important for historical studies (e.g., Ogburn, 2006; Sevenant & Antrop, 2007) and has found application also in a number of areas of current interest, such as seeking locations to place objects potentially harming scenic beauty like photovoltaic power plants or wind farms (Fernandez-Jimenez et al., 2015; Sklenicka & Zouhar, 2018), coastal aquaculture sites (Falconer et al., 2013), and ski areas (Geneletti, 2008); placing military structures (Smith & Cochrane, 2011); tagging landscape photographs (Brabyn & Mark, 2011); analyzing the effects of introducing animal species (Kizuka et al., 2014); and modelling predation risk in animal ecology (Alonso, Álvarez Martínez & Palacín, 2012; Olsoy et al., 2015). The basic algorithm implemented in most GIS software produces a binary detection of areas that are visible or nonvisible from a point of observation or identifies areas from which a given object in the landscape is or is not visible. Combining several such binary viewsheds created from multiple observation points or from all cells in the raster of the study area creates a cumulative viewshed describing the visual exposure of the study area. As a number of factors may play roles in visibility modelling and using only a binary attribute (0 or 1) constitutes a drastic simplification (Fisher, 1992), other algorithms have been under development for a number of years, such as fuzzy viewshed and visual magnitude (Chamberlain & Meitner, 2013; Fernandez-Jimenez et al., 2015; Fisher, 1992; Fisher, 1993; Fisher, 1994; Fisher, 1995; Fisher, 1996; Ogburn, 2006), as well as visibility indices such as the Vertical Visibility Index (Nutsford et al., 2015), which enrich the model with further parameters and so are used to bring it closer to reality. Due to their simplicity and implementation in common GIS software, however, binary and cumulative viewsheds are still used in a number of studies (e.g., Alonso, Álvarez Martínez & Palacín, 2012; Falconer et al., 2013; La Rosa, 2011; Olsoy et al., 2015; Schirpke, Tasser & Tappeiner, 2013).

In addition to the computational algorithm, the reliability of the resulting visibility model also depends on the quality of the input digital surface model (DSM) (Klouček, Lagner & Šímová, 2015; Lake et al., 2000; Sander & Manson, 2007), and Fisher (1992) previously noted that it would be an error to assume the input DSM to be accurate. Although many studies have dealt with improving algorithms, only a few studies have focused on the effect the spatial accuracy of input data has on the reliability of results from visibility analyses, even though, as can been seen in older visibility studies (Fisher, 1992; Huss & Pumar, 1997) and spatial uncertainty research in other areas (for review see Barry & Elith, 2006; Moudrý & Šímová, 2012), it is highly probable that decreased data quality correlates with decreased quality of results. DSMs for visibility analyses are mostly created as combinations of digital terrain models (DTMs) depicting the bare earth surface plus layers of objects on that surface, particularly structures and vegetation. As such layers rarely contain the attribute object height, the height for creating the DSM is estimated based on knowledge of the area or such sources as published works on vegetation in the location, as was done by Schirpke, Tasser & Tappeiner (2013). The accuracy of this estimate represents an additional potential source of DSM inaccuracy beyond the scale of elevation and planimetric data. In extreme cases, objects are entirely omitted from the surface and visibility is modelled based only upon a DTM, even though Dean (1997) has already demonstrated the logical expectation that using DSM results in higher-quality visibility models.

Examples of rare studies dealing with input data precision have been presented by Lake et al. (2000) and Sander & Manson (2007), who focused upon modelling structures as vertical obstacles to visibility. Some authors have focused on modelling vegetation for visibility analysis, but they did not evaluate the effect of such models’ precision on the precision of the visibility model (e.g., Domingo-Santos et al., 2011). Problems with implementing vegetation and structures into DSMs do not arise when using LiDAR-based surface models, which already contain objects on the surface and are considered by many authors to be currently the most accurate data input for visibility analyses (see Castro, García-Espona & Iglesias, 2015; Lake et al., 2000; Murgoitio et al., 2014). Using the example of wind turbine visibility and comparing modelled visibility with actual visibility in the field, Klouček, Lagner & Šímová (2015) demonstrated that use of a LiDAR-based DSM can result in an approximately 90% match rate with reality while the use of DSMs based on vector layers of various scales resulted in only 50–80% match rates. Unfortunately, LiDAR-based DSMs cannot yet be used for all study areas due to their high prices, because of the difficulty in processing a point cloud into a raster DSM, and not least for the reason that LiDAR data is not yet available for a number of areas. For these reasons, it is necessary to know how visibility models based on other data differ from LiDAR-based models and whether these differences depend on the quality of the vegetation height estimation and other variables.

The aim of our study was to evaluate the effect of the quality of DSMs (which are conditioned by the quality of DTM data and by tree heights expertly assigned during DSM construction) on the accuracy of a simple binary viewshed analysis. We hypothesized that models with lower resolution will lead to higher overestimations of the viewshed.

## Methods

### Sampling locations

**Figure 1:**
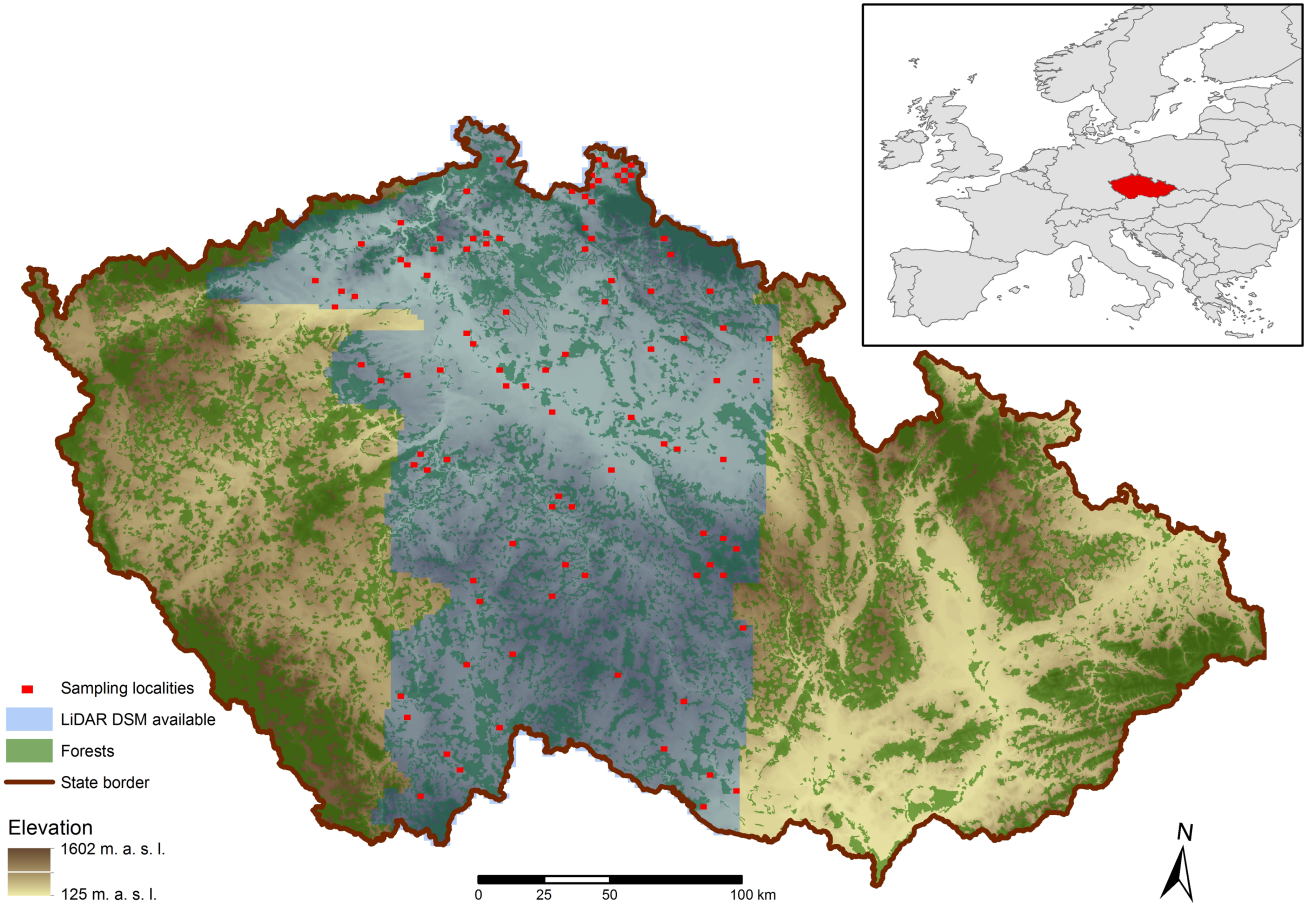
Sampling locations.

We analyzed visibility at 104 sampling locations in the Czech Republic. One location corresponded to a single page of a national map at a scale of 1:5,000 (i.e., a rectangle of 2.5 × 2 km). Selecting locations in this manner provided sufficient areas for visibility analyses at a detailed scale while still enabling acquisition of input data for a sufficient number of locations. The locations (map pages) were selected by stratified random sampling from that section of the Czech Republic which had available DTMs as well as DSMs created from airborne laser scanning data. This section forms a north–south band in the center of the country (Fig. 1) covering elevations ranging from lowlands to mountains (141 to 928 m a.s.l.) and various landscape types from agricultural to forest. Random sampling of locations was stratified so that it would include as equally as possible combinations of variously forested areas (three categories according to the proportion of forest at the location: 0–9%, 10–24%, and 25–60%; the proportions were computed from vector map at a 1:25,000 scale) and various terrain configurations (three categories according to elevation differences in the area expressed as the standard deviation of elevation in the location: <10, 11–30, >30 m; the values were computed from MAP25). Another condition was excluding selection of adjacent map pages.

### Input data and GIS processing

All GIS analyses were conducted using ArcGIS 10.2 software (ESRI, CA, USA). For all viewshed analyses, we used five input DSMs varying in scale and accuracy (see Table 1 for overview). The most accurate was the 1st Generation LiDAR-based DSM of the Czech Republic (hereinafter LiDAR). It was also the only dataset that was available directly as a DSM for the sampling locations. The remaining DSMs were created as sums of rasters comprising the terrain (DTMs) and objects on the terrain (digital object models [DOMs]). Working in this manner, one of the inputs combined a LiDAR-based DTM with a vectorization of forests and built-up areas on the actual orthophotomap (hereinafter LidOrth). The remaining DSMs were based on vector topographic maps at scales of 1:5,000 (hereinafter MAP5), 1:25,000 (MAP25), and 1:500,000 (MAP500) (see Table 2 for overview). In all these datasets, elevation was depicted by contour lines and topography by polygons representing the footprints of individual objects on the ground. DTMs were calculated by interpolating contour lines using the topo to raster method. To create DOM rasters, we added estimated values of heights to polygons of visual obstacles and rasterized the layers. Inasmuch as forests were the most important visual obstacles within the locations, we tested five values of forest height (15, 20, 25, 30, and 35 m) to evaluate the effect of the DOMs’ height estimates on viewshed results. These values represent a range of mature forest types under various ecological conditions in the Czech Republic. Other woody vegetation types, such as young forests and orchards, were assigned the height of 5 m. We assigned the height of 8 m to buildings and built-up areas as an estimate of the average height of rural structures within the locations.

**Table 1:** Description of input datasets.

Acronym within study	Czech acronym	Scale	Year of last update	Elevation accuracy	Planimetric accuracy	Contour interval	Data description
LiDAR	DMP 1G	Density of elevation point cloud is 1–2 points/m^2^	2009–2013	0.4–0.7 m	0.4–0.7 m	No contour	Digital surface model represented by elevation point cloud from data acquired by aerial LiDAR covering part of the Czech Republic
LidOrth	DMR 5G	Density of elevation point cloud is 1–2 points/m^2^	2009–2013	0.18–0.3 m	Only elevation dataset	No contour	Digital terrain model represented by elevation point cloud from data acquired by aerial LiDAR covering part of the Czech Republic
	Orthophotomap	Pixel resolution =0.5 m	2013	Only planimetric dataset	0.25–0.5 m		Orthophotomap covering the entire Czech Republic
Map5	SM 5	1:5,000	2001–2014	0.7–5 m	0.5–1 m	1, 2, or 5 m depending on the character of the terrain	Large-scale vector database covering part of the Czech Republic
Map25	DMU 25	1:25,000	1998	5–10 m	0.5–20 m	5 m	Medium-scale vector database covering the entire Czech Republic
Map500	ArcCR 500	1:500,000	2014	25–50 m	Up to 200 m	50 m	Small-scale vector database covering the entire Czech Republic

**Table 2:** Creation of five digital surface models (DSMs) from input datasets.

DSM		DTM—source elevation data		**DOM—source planimetric data**
LiDAR	=	elevation point cloud	=	elevation point cloud
LidOrth	=	elevation point cloud	+	vectorization on actual orthophotomap: forest (15–35 m), orchard (5 m), built-up area (8 m)
MAP5	=	MAP5 (contour lines)	+	Map5: forest (15–35 m), orchard (5 m), built-up area (8 m)
MAP25	=	MAP25 (contour lines)	+	Map25: forest (15–35 m), orchard (5 m), built-up area (8 m)
MAP500	=	MAP500 (contour lines)	+	MAP500: forest (15–35 m), built-up area (8 m)

The ArcGIS Viewshed tool, which creates simple binary layers distinguishing between visible and nonvisible areas, was employed for GIS visibility analyses and the process was automatized using a Python script. Within each sampling location, we generated one random point outside of the forested areas as the observer location. We processed a set of viewshed analyses with all of the DSMs and with two heights assigned to the observer point as the OFFSETA parameter within the Viewshed tool (the OFFSETA parameter simulates observer’s height—it is a vertical distance that is added to the vertical value of the cell. The height of 1.8 m simulated observation of the landscape by a person standing on the ground (*ground* variant—1.8 m above terrain). The second variant used the height of 80 m, which can be interpreted as visibility from an observation tower or as visibility from a tall structure such as a wind turbine in the landscape (approximately, disregarding height of the observer; *tower* variant). In this way, we created 2 × 21 viewshed models, i.e., binary rasters (visible-not visible) for each sampling location.

### Statistical analysis

We used an R (R Core Team, 2015) script for the nonparametric Friedman’s ANOVA with repeated measures design and post-hoc test (available from http://www.r-statistics.com/2010/02/post-hoc-analysis-for-friedmans-test-r-code/) to analyze potential differences among visibilities modelled with different forest heights. The response variable was the amount of visible area as a percentage of the location obtained from the viewshed models for each dataset and the forests heights were designed as repeated measures at the same location. The identical procedure was used to analyze differences among visibilities obtained from individual datasets. In this case, the response variable was the percentage of visible area modelled with the forest height of 25 m and the datasets were taken as repeated measures at the location. Similarly, we used this design and the Friedman test to test the significance of spatial differences among modelled visibilities. In accordance with previous studies (Castro, García-Espona & Iglesias, 2015; Klouček, Lagner & Šímová, 2015; Lake et al., 2000; Murgoitio et al., 2014), we considered the model based on the LiDAR DSM to be the most accurate (as best matching reality). Hence, the response variable was calculated as the spatial difference (Symmetrical Difference Tool in ArcGIS) between LiDAR visibility and visibility modeled with an individual dataset (LiDAR vs. LidOrth, LiDAR vs. MAP5, LiDAR vs. MAP25 and LiDAR vs. MAP500). In comparison with a simple numerical subtraction, spatial difference reflects also the cases, when visible areas are numerically similar but their shape and location differ.

## Results

**Figure 2:**
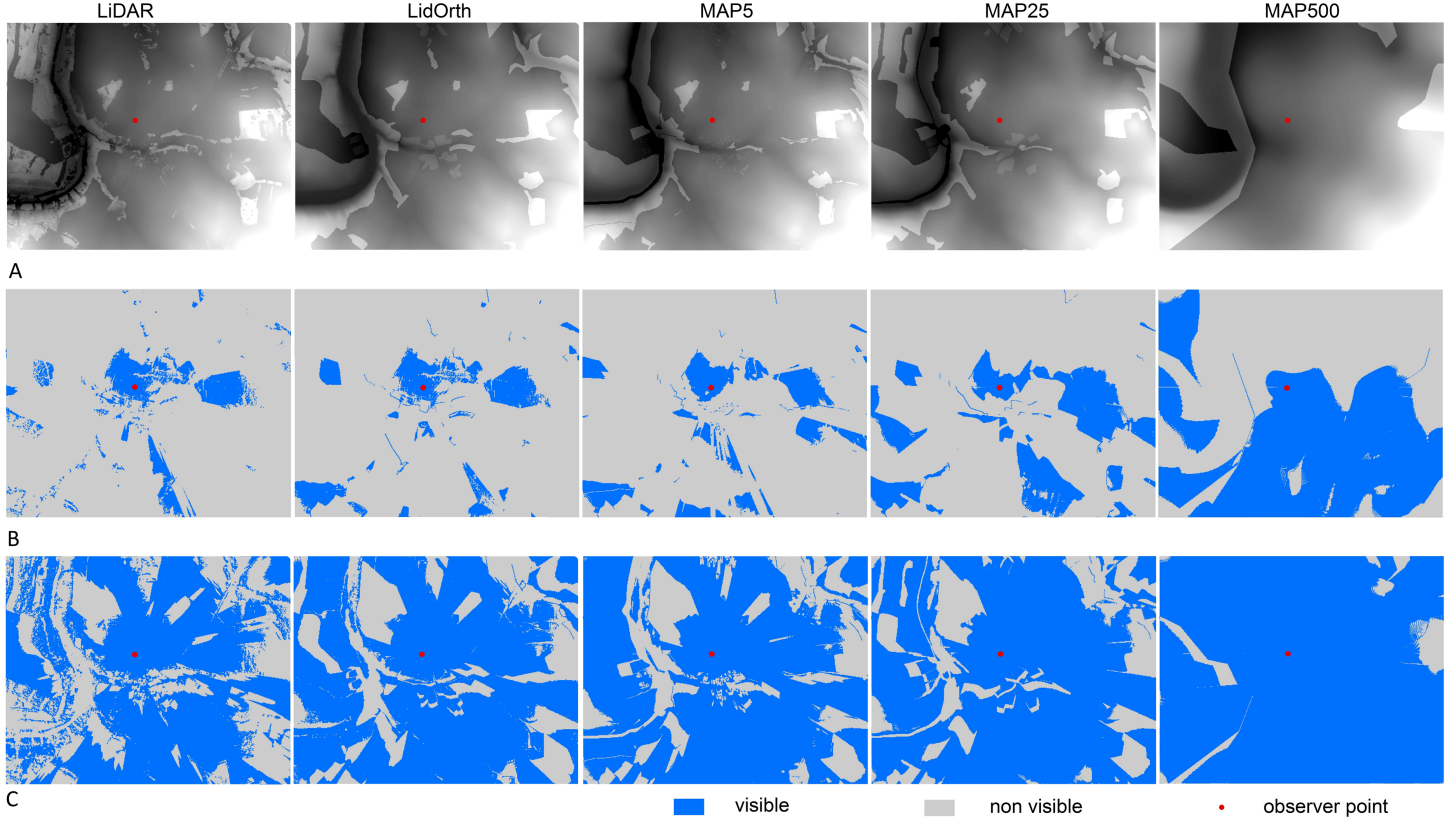
Overestimation of visible area depending on input DSM scale and observer point height—an example of one sampling location (A) Digital surface model. (B) Visibility model—ground variant. (C) Visibility model—tower variant.

As can be seen in Table 3, using a tower as the observation point or observed object leads, as expected, to larger viewsheds modelled based on each dataset in comparison to the area visible to a ground-level observer, although the trend of differences among datasets is similar for both observer point heights. The smallest average size of visible area in sampling locations came from LiDAR model, while all of the remaining datasets led to considerable overestimations in the resulting viewshed (see Fig. 2 for an illustration). For both observer point heights, the viewshed size resulting from LiDAR model (on average 6.76% of the location when observing from the ground and 51.40% from 80 m) clearly differed from the sizes calculated using the other datasets. For the ground-level variant, the results closest to those of the LiDAR model were achieved by the model based on LidOrth, followed by the models based on MAP25 and MAP5, which had average visibility similar to one another. For the observer point height of 80 m, there were minimal differences among results acquired using LidOrth, MAP5, and MAP25. For both variants, the model based on MAP500 produced the largest viewshed overestimations. For ground-level observation, the LidOrth model produced visible areas approximately 70% larger than those produced by the LiDAR model. The MAP5- and MAP25 models resulted in visible areas more than twice as large and the MAP500-based model more than five times as large as those produced by the LiDAR model. Although the differences in visible area did not come to such large multiples for the tower variant, the visibility modelled based on various datasets differed by more percentage points and the differences therefore concerned a larger proportion of the area. The LidOrth-, MAP5-, and MAP25-based models produced visible areas almost 20 percentage points larger than did the LiDAR-based model, and the MAP500-based model produced visible areas about 30 percentage points larger (Table 3). This simple overview of percentages also corresponds to the results of the Friedman test for models using a forest height of 25 m (Table 4). Significant differences in visibility modelled based on the MAP5 and MAP25 datasets were not recorded for any of the observer point heights. In addition, there was no significant difference for the tower variant between the LidOrth-based and MAP25-based models. All remaining differences among datasets were significant, and frequently very highly so (see Table 4).

**Table 3:** Sizes of visible area as a percent of the sampling location standard deviation modelled on basis of individual datasets for observation from ground level (ground) and from a height of 80 m (tower).

		Forest height				
DSM	Level	15 m	20 m	25 m	30 m	35 m
LiDAR	*Ground*	–	–	6.76 ± 6.88	–	–
	*tower*	–	–	51.40 ± 16.89	–	
LidOrth	*ground*	12.04 ± 10.07	11.75 ± 9.90	11.50 ± 9.77	11.32 ± 9.68	11.29 ± 9.64
	*tower*	71.46 ± 15.64	69.80 ± 15.80	68.16 ± 16.03	66.56 ± 16.31	65.01 ± 16.66
MAP5	*ground*	16.35 ± 12.85	16.01 ± 12.76	15.72 ± 12.73	15.49 ± 12.71	15.34 ± 12.66
	*tower*	73.59 ± 15.57	71.92 ± 15.74	70.27 ± 16.02	68.65 ± 16.34	67.06 ± 16.71
MAP25	*ground*	16.18 ± 13.92	15.48 ± 13.71	14.97 ± 13.63	14.56 ± 13.53	14.24 ± 13.41
	*tower*	72.36 ± 15.94	70.12 ± 16.26	67.95 ± 16.74	65.89 ± 17.25	64.00 ± 17.56
MAP500	*ground*	37.33 ± 22.61	36.74 ± 22.53	36.20 ± 22.47	35.74 ± 22.43	35.36 ± 22.41
	*tower*	86.37 ± 14.60	85.29 ± 14.97	84.22 ± 15.49	83.18 ± 16.13	82.25 ± 16.65

**Table 4:** Significance of size differences among visible areas modelled based on individual datasets using a forest height of 25 m for *ground* and *tower* variants. Friedman test with repeated measures design and post-hoc test. Significant values are in bold.

	LidOrth		MAP5		MAP25		MAP500	
	*ground*	*tower*	*ground*	*tower*	*ground*	*tower*	*ground*	*tower*
LiDAR	<**0.002**	<**1e**^−**9**^	<**1e**^−**16**^	<**1e**^−**16**^	<**1e**^−**11**^	<**1e**^−**16**^	<**1e**^−**16**^	<**1e**^−**16**^
LidOrth			<**2e**^−**5**^	<**0.0002**	<**0.01**	0.051	<**1e**^−**16**^	<**1e**^−**16**^
MAP5					0.554	0.455	<**1e**^−**9**^	<**1e**^−**9**^
MAP25							<**5e**^−**14**^	<**1e**^−**14**^

Spatial differences between visibilities based on individual datasets and LiDAR-based visibility (see Table 5) were larger than the differences acquired through numerically subtracting visible areas, although numerical and spatial differences displayed the same trend (the Spearman correlation coefficient for numerical and spatial differences varied between 0.845 and 0.957). In terms of spatial differences, the LidOrth-based model differed in resulting visibility from the LiDAR-based model by 8.05 percentage points in the *ground* variant and by 25.75 percentage points in the *tower* variant. The spatial differences between other models and the LiDAR-based model were significantly greater than was that for the LidOrth-based model (see Table 6 for *p*-values). The differences between the remaining datasets and the LiDAR-based model expressed as percentage points were (Model: *ground*, *tower*): MAP5: 12.52, 26.56; MAP25: 12.44, 26.62; and MAP500: 32.5, 35.29. Similarly as for the analyses focused on total visible area (Table 4), the spatial difference analysis also resulted in no significant differences from the LiDAR-based model for visibilities calculated based on MAP5 and MAP25 (Table 6). Visibility based on MAP500 again very significantly differed from that based on all of the others.

**Table 5:** Overestimations by individual models when compared to LiDAR results.

Model based on:	LiDAR	LidOrth	MAP5	MAP25	MAP500
Difference Ground (1.8 m)	0	8.05	12.52	12.44	32.5
Difference Tower (80 m)	0	25.75	26.56	26.62	35.29

**Table 6:** Significance of spatial differences among modelled visibilities. The response variable was calculated as the spatial difference between LiDAR visibility and the visibility modeled by an individual dataset. Friedman test with repeated measures design and post-hoc test. Significant values are in bold.

	MAP5		MAP25		MAP500	
	*ground*	*tower*	*ground*	*tower*	*ground*	*tower*
LidOrth	<**5e**^−**8**^	<0.005	<**5e**^−**7**^	<**0.0001**	<**1e**^−**16**^	<**1e**^−**16**^
MAP5			0.998	0.852	<**5e**^−**16**^	<**1e**^−**16**^
MAP25					<**1e**^−**16**^	<**5e**^−**15**^

In general, it can be concluded from the analysis as to effect of dataset used on resulting visibility that viewshed models calculated using a combination of a LiDAR-based DTM with vectorization on an orthophotomap (LidOrth) provide similar results as do models created based on maps at scales 1:5,000 to 1:25,000, although modelled visibility is strongly overestimated in comparison to models based on LiDAR-based DSMs.

Looking at the effect of forest height (Table 3), it is apparent that the visible area decreases with taller forest height, although the effect is much smaller than that caused by the quality of the terrain model. Other effects of forest height are demonstrated in the LidOrth dataset, representing datasets giving similar visible area values (LidOrth, MAP5, and MAP25), and the MAP500 dataset as the dataset giving the most different results (see Table 7). For the *ground* variant, visible area extent was in most cases significantly different when the forest height was changed by 10 m, while a change of 5 m was sufficient in the *tower* variant. All of the heights produced results significantly different from those of the LiDAR-based model. The significance of all of the differences had a decreasing tendency with coarser scale and tended to be lower for the *ground* variant than for the *tower* variant. For the combination of all effects, the difference from the LiDAR-based model was least apparent for the *ground* model with MAP500 as the input dataset and forest height of 35 m (*p* = 0.048). Given the overall overestimation of visibility by all datasets, however, it cannot be stated that the tallest forest height estimate is the most suitable for calculating viewshed. Despite their statistical significance, percentage differences in visible area size caused by changes in forest height were minimal in comparison to those caused by input data accuracy. It can therefore be stated that the effect of data detail on modelled visibility is dominant and that when using surfaces not based on LiDAR object height accuracy has only a secondary effect on the accuracy of the result.

**Table 7:** Significance of size differences among visible areas for LiDAR-based DSM and different forest heights for *ground* and *tower* variants. *P*-values are presented for one of those datasets with similar results (LidOrth) and the most different dataset (MAP500). Friedman test with repeated measures design and post-hoc test. Significant values are in bold.

		15 m		20 m		25 m		30 m		35 m	
		*ground*	*tower*	*ground*	*tower*	*ground*	*tower*	*ground*	*tower*	*ground*	*tower*
LiDAR	LidOrth	<**1e**^−**16**^	<**1e**^−**16**^	<**1e**^−**16**^	<**1e**^−**16**^	<**5e**^−**11**^	<**1e**^−**16**^	<**1e**^−**4**^	<**5e**^−**12**^	<**0.05**	**0.005**
	MAP500	<**1e**^−**16**^	<**1e**^−**16**^	<**1e**^−**16**^	<**1e**^−**16**^	<**1e**^−**16**^	<**1e**^−**16**^	<**1e**^−**16**^	<**1e**^−**16**^	<**5e**^−**14**^	<**5e**^−**7**^
15 m	LidOrth			0.524	<**0.01**	<**5e**^−**4**^	<**5e**^−**12**^	<**5e**^−**12**^	<**1e**^−**16**^	<**1e**^−**15**^	<**1e**^−**16**^
	MAP500			0.219	<**0.05**	<**5e**^−**4**^	<**5e**^−**9**^	<**5e**^−**10**^	<**1e**^−**16**^	<**5e**^−**16**^	<**1e**^−**16**^
20 m	LidOrth					0.127	<**0.005**	<**5e**^−**7**^	<**5e**^−**12**^	<**5e**^−**10**^	<**1e**^−**16**^
	MAP500					0.257	<**0.05**	<**5e**^−**4**^	<**5e**^−**9**^	<**5e**^−**9**^	<**1e**^−**16**^
25 m	LidOrth							<**0.05**	<**0.005**	<**0.001**	<**5e**^−**12**^
	MAP500							0.238	<**0.05**	<**5e**^−**4**^	<**5e**^−**10**^
30 m	LidOrth									0.931	<**0.005**
	MAP500									0.322	<**0.01**

How spatial differences between the visibility modelled with a given dataset and LiDAR-based visibility depended on terrain configuration and number of obstacles cannot be generalized, because individual datasets in combination with the *ground* and *tower* variants produced varying results.

## Discussion

This study compares the results of visibility models based on data of various spatial accuracy with models based on a LiDAR-based dataset, the latter of which most closely matches reality according to the field comparison of modelled visibility by Klouček, Lagner & Šímová (2015). We expected that the models would be overestimating the LiDAR-based viewsheds as Lidar provides a full digital surface model including all low vegetation or obstacles that cannot be properly captured when using any of the other models. We also expected that the LidOrth model would be the best of the composite models as the terrain model is very accurate and is not subject to generalization as in the case of MAP-based models. Our results confirmed that assumption, all of the other models considerably overestimated visibility in comparison to the LiDAR-based model.

We had also expected that, with the exception of the LidOrth dataset combining a LiDAR-based DTM with objects digitized on an actual orthophotomap, the smallest difference would appear in visibility modelled on basis of the most detailed vector data (i.e., MAP5). Surprisingly, however, MAP5 provided similar results as did MAP25, whether working with numerical or spatial differences in visible areas. In addition to the fact that MAP5 is at a more detailed scale than is MAP25, MAP5 is the only tested dataset that depicts individual buildings and not just outlines of built-up areas. In accordance with the results of Sander & Manson (2007), who stated that generalizing building locations has a significant effect on the resulting viewshed model and that this effect is more important than is that from imprecise building height determination, we predicted that MAP5 would produce a more precise viewshed model. However, Sander & Manson (2007) analyzed visibility in cities and our results indicate that in locations in the countryside, where buildings occur to a lesser extent and are predominantly part of smaller municipalities as in our study, then generalizing buildings does not have a significant effect on visibility modelling results. For all variants evaluated, visibility modelled on the basis of MAP500 differed the most from the other visibility models. This result corresponds to the low reliability of visibility models based on this dataset (48.1–63.9%) found by Klouček, Lagner & Šímová (2015). Data generalized to such an extent as is found in maps at a scale of 1:500,000 therefore cannot be used at such a detailed scale (areas of 5 km^2^) for modelling visibility, not even when objects on the surface are included from the planimetric layers of such a map. The fact that even LidOrth, which utilizes the same terrain as LiDAR model, showed overestimation, leads to a conclusion that the expertly assigned tree heights were indeed the principal source of the error. In some observer points, however, even omission of taller herbaceous vegetation could have caused an overestimation for the ground variant as it might have partly obstructed the “observers” view.

In relation to the findings of this study and those of Klouček, Lagner & Šímová (2015), it can be difficult to understand the results of studies that do not describe in detail the input data used to model visibility. This is a problem for certain applied studies that do not have as their primary objective to study the effect of geodata on the results. For example, Geneletti (2008) modelled the visibility of ski areas in a range of 5 km, which means within the zone of greatest visual effect (e.g., Betakova, Vojar & Sklenicka, 2015), based on a DSM, but that author did not state how and from what data the DSM was assembled. Etherington & Alexander (2008) stated the scale of the digital elevation model (1:20,000) and the resolution of the raster (30 m) used for their viewshed model, but it is not clear whether this raster included vegetation. Given that the scale of data used has a dominant effect on visibility results, all future studies should describe the input data so that the applicability of the study results can be evaluated.

According to our results, it cannot be stated unequivocally that the rate of spatial overestimation by datasets would be, say, higher in flat or mountainous terrain or in areas that are more or less forested. Our work considered obstacles to visibility to be opaque. This is not necessarily the case, however, and particularly not in the case of forest stands. Therefore, searches are underway for techniques to model forests more realistically than as solid polygons with uniform tree height (e.g., Domingo-Santos et al., 2011; Liu et al., 2010). Such forest models work with individual trees and thereby take into account both stand density and set crown height, with stands having crown height set higher being more transparent. Our results indicate, however, that at the given evaluation scale (locations of 5 km^2^) such labor-intensive modelling of stands is not significant for the results, as the effect of input data scale is dominant. This can be seen in the fact that all of the datasets used produced overestimations in comparison to the LiDAR-based model. Making forest stands transparent would result in a higher percentage of visible area at a given location (i.e., even greater overestimations) and thus increasing the accuracy of obstacle models would paradoxically further add to viewshed model inaccuracy.

The LiDAR-based DSMs used in this study originate from nationwide imaging which did not have as its primary objective to create DSMs in non-built-up areas. The fact that the imaging took place also outside of the growing season can, together with the low point cloud density, lead to inaccuracy in the DSMs, particularly in places with broadleaf vegetation. It is therefore possible that use of more detailed LiDAR captured during the growing season would reveal even greater spatial overestimation of visibility by all tested datasets.

## Conclusions

This comparison of visibilities modelled using the LiDAR-based DSM and DSMs based on vector datasets or on a combination of the LiDAR DTM and an orthophotomap indicates that all of the other models considerably overestimated visibility in comparison to the LiDAR-based model. The overestimation rate was greater in absolute numbers with a higher observer point, although trends in overestimations were identical in models simulating observation from the ground and those simulating observation from a tower. In both cases, it can be stated that none of the other datasets with any set height for obstacles to visibility approached the accuracy of the LiDAR-based visibility model and that the established obstacle height had a minor effect on resulting visibility in comparison to the effect of the dataset.

## Additional Information and Declarations

### Data Availability

The following information was supplied regarding data availability: Lagner, Ondřej (2018): data_PeerJ.zip. figshare. Fileset. https://doi.org/10.6084/m9.figshare.6199961.v1.
